# A Nested Case–Control Study of Metabolically Defined Body Size Phenotypes and Risk of Colorectal Cancer in the European Prospective Investigation into Cancer and Nutrition (EPIC)

**DOI:** 10.1371/journal.pmed.1001988

**Published:** 2016-04-05

**Authors:** Neil Murphy, Amanda J. Cross, Mustapha Abubakar, Mazda Jenab, Krasimira Aleksandrova, Marie-Christine Boutron-Ruault, Laure Dossus, Antoine Racine, Tilman Kühn, Verena A. Katzke, Anne Tjønneland, Kristina E. N. Petersen, Kim Overvad, J. Ramón Quirós, Paula Jakszyn, Esther Molina-Montes, Miren Dorronsoro, José-María Huerta, Aurelio Barricarte, Kay-Tee Khaw, Nick Wareham, Ruth C. Travis, Antonia Trichopoulou, Pagona Lagiou, Dimitrios Trichopoulos, Giovanna Masala, Vittorio Krogh, Rosario Tumino, Paolo Vineis, Salvatore Panico, H. Bas Bueno-de-Mesquita, Peter D. Siersema, Petra H. Peeters, Bodil Ohlsson, Ulrika Ericson, Richard Palmqvist, Hanna Nyström, Elisabete Weiderpass, Guri Skeie, Heinz Freisling, So Yeon Kong, Kostas Tsilidis, David C. Muller, Elio Riboli, Marc J Gunter

**Affiliations:** 1 Department of Epidemiology and Biostatistics, School of Public Health, Imperial College London, London, United Kingdom; 2 Division of Genetics and Epidemiology, Institute of Cancer Research, Sutton, United Kingdom; 3 International Agency for Research on Cancer, World Health Organization, Lyon, France; 4 Department of Epidemiology, German Institute of Human Nutrition Potsdam-Rehbruecke, Potsdam, Germany; 5 Inserm, Nutrition, Hormones and Women’s Health, Centre for Research in Epidemiology and Population Health (CESP), U1018, Villejuif, France; 6 Université Paris Sud, UMRS 1018, Villejuif, France; 7 Institut Gustave Roussy, Villejuif, France; 8 Division of Cancer Epidemiology, German Cancer Research Center (DKFZ), Heidelberg, Germany; 9 Danish Cancer Society Research Center, Copenhagen, Denmark; 10 Section for Epidemiology, Department of Public Health, Aarhus University, Aarhus, Denmark; 11 Public Health Directorate, Asturias, Spain; 12 Unit of Nutrition, Environment and Cancer, Catalan Institute of Oncology, Barcelona, Spain; 13 Andalusian School of Public Health, Granada, Spain; 14 Biomedical Research Centre Network for Epidemiology and Public Health (CIBERESP), Madrid, Spain; 15 Public Health Direction and Biodonostia–CIBERESP, Basque Regional Health Department, Vitoria, Spain; 16 Department of Epidemiology, Murcia Regional Health Council, Murcia, Spain; 17 Navarre Public Health Institute, Pamplona, Spain; 18 University of Cambridge, Cambridge, United Kingdom; 19 MRC Epidemiology Unit, Cambridge, United Kingdom; 20 Cancer Epidemiology Unit, Nuffield Department of Population Health, University of Oxford, Oxford, United Kingdom; 21 Hellenic Health Foundation, Athens, Greece; 22 Department of Hygiene, Epidemiology and Medical Statistics, University of Athens Medical School, Athens, Greece; 23 Bureau of Epidemiologic Research, Academy of Athens, Athens, Greece; 24 Department of Epidemiology, Harvard School of Public Health, Boston, Massachusetts, United States of America; 25 Molecular and Nutritional Epidemiology Unit, Cancer Research and Prevention Institute (ISPO), Florence, Italy; 26 Epidemiology and Prevention Unit, Fondazione IRCCS Istituto Nazionale dei Tumori, Milan, Italy; 27 Cancer Registry and Histopathology Unit, Civic–M.P.Arezzo Hospital, Azienda Sanitaria Provinciale di Ragusa, Italy; 28 HuGeF Foundation, Torino, Italy; 29 Dipartimento di Medicina Clinica e Sperimentale, Federico II University, Naples, Italy; 30 Department of Determinants of Chronic Diseases, National Institute for Public Health and the Environment (RIVM), Bilthoven, The Netherlands; 31 Department of Gastroenterology and Hepatology, University Medical Centre Utrecht, Utrecht, The Netherlands; 32 Department of Social & Preventive Medicine, Faculty of Medicine, University of Malaya, Kuala Lumpur, Malaysia; 33 Julius Center for Health Sciences and Primary Care, University Medical Center Utrecht, Utrecht, The Netherlands; 34 Division of Internal Medicine, Department of Clinical Sciences, Skåne University Hospital, Lund University, Malmö, Sweden; 35 Diabetes and Cardiovascular Disease–Genetic Epidemiology, Department of Clinical Sciences, Skåne University Hospital, Lund University, Malmö, Lund University, Sweden; 36 Medical Bioscience, Umeå University, Umeå, Sweden; 37 Department of Community Medicine, Faculty of Health Sciences, University of Tromsø–The Arctic University of Norway, Tromsø, Norway; 38 Cancer Registry of Norway, Oslo, Norway; 39 Department of Medical Epidemiology and Biostatistics, Karolinska Institutet, Stockholm, Sweden; 40 Department of Genetic Epidemiology, Folkhälsan Research Center, Helsinki, Finland; 41 Department of Hygiene and Epidemiology, University of Ioannina School of Medicine, Ioannina, Greece; Harvard Medical School, UNITED STATES

## Abstract

**Background:**

Obesity is positively associated with colorectal cancer. Recently, body size subtypes categorised by the prevalence of hyperinsulinaemia have been defined, and metabolically healthy overweight/obese individuals (without hyperinsulinaemia) have been suggested to be at lower risk of cardiovascular disease than their metabolically unhealthy (hyperinsulinaemic) overweight/obese counterparts. Whether similarly variable relationships exist for metabolically defined body size phenotypes and colorectal cancer risk is unknown.

**Methods and Findings:**

The association of metabolically defined body size phenotypes with colorectal cancer was investigated in a case–control study nested within the European Prospective Investigation into Cancer and Nutrition (EPIC) study. Metabolic health/body size phenotypes were defined according to hyperinsulinaemia status using serum concentrations of C-peptide, a marker of insulin secretion. A total of 737 incident colorectal cancer cases and 737 matched controls were divided into tertiles based on the distribution of C-peptide concentration amongst the control population, and participants were classified as metabolically healthy if below the first tertile of C-peptide and metabolically unhealthy if above the first tertile. These metabolic health definitions were then combined with body mass index (BMI) measurements to create four metabolic health/body size phenotype categories: (1) metabolically healthy/normal weight (BMI < 25 kg/m^2^), (2) metabolically healthy/overweight (BMI ≥ 25 kg/m^2^), (3) metabolically unhealthy/normal weight (BMI < 25 kg/m^2^), and (4) metabolically unhealthy/overweight (BMI ≥ 25 kg/m^2^). Additionally, in separate models, waist circumference measurements (using the International Diabetes Federation cut-points [≥80 cm for women and ≥94 cm for men]) were used (instead of BMI) to create the four metabolic health/body size phenotype categories. Statistical tests used in the analysis were all two-sided, and a *p*-value of <0.05 was considered statistically significant. In multivariable-adjusted conditional logistic regression models with BMI used to define adiposity, compared with metabolically healthy/normal weight individuals, we observed a higher colorectal cancer risk among metabolically unhealthy/normal weight (odds ratio [OR] = 1.59, 95% CI 1.10–2.28) and metabolically unhealthy/overweight (OR = 1.40, 95% CI 1.01–1.94) participants, but not among metabolically healthy/overweight individuals (OR = 0.96, 95% CI 0.65–1.42). Among the overweight individuals, lower colorectal cancer risk was observed for metabolically healthy/overweight individuals compared with metabolically unhealthy/overweight individuals (OR = 0.69, 95% CI 0.49–0.96). These associations were generally consistent when waist circumference was used as the measure of adiposity. To our knowledge, there is no universally accepted clinical definition for using C-peptide level as an indication of hyperinsulinaemia. Therefore, a possible limitation of our analysis was that the classification of individuals as being hyperinsulinaemic—based on their C-peptide level—was arbitrary. However, when we used quartiles or the median of C-peptide, instead of tertiles, as the cut-point of hyperinsulinaemia, a similar pattern of associations was observed.

**Conclusions:**

These results support the idea that individuals with the metabolically healthy/overweight phenotype (with normal insulin levels) are at lower colorectal cancer risk than those with hyperinsulinaemia. The combination of anthropometric measures with metabolic parameters, such as C-peptide, may be useful for defining strata of the population at greater risk of colorectal cancer.

## Abstract 2

Gunter and colleagues analyse a large European dataset to determine how body size and metabolic profile associates with the risk of developing colorectal cancer.

## Editors' Summary

**Background:**

Colorectal cancer is the third most common cancer worldwide and is a leading cause of cancer-related death, killing around 700,000 people every year. It develops when cells in the colon (the final part of the digestive system, which is also known as the large intestine or large bowel) or the rectum (the lower end of the colon) acquire genetic changes that allow them to divide uncontrollably to form a tumor and to move around the body (metastasize). Symptoms of colorectal cancer include blood in the stool, a change in bowel habits, and unexplained weight loss. Treatments for colorectal cancer include surgery, chemotherapy, and radiation. As with other types of cancer, these treatments are more likely to be successful if started when the tumor is very small. Consequently, many countries run screening programs that use colonoscopy, the fecal occult blood test, and other tests to detect the earliest signs of colorectal cancer in apparently healthy people.

**Why Was This Study Done?:**

Being obese—having too much body fat—is associated with an increased colorectal cancer risk (other risk factors include age, having a family history of colorectal cancer, and eating a high-fat, low-fiber diet). Obesity is also associated with several other chronic diseases, and recent evidence suggests that some obese individuals have a higher risk of developing these diseases than others. For example, overweight/obese individuals who have hyperinsulinemia (abnormally high blood levels of insulin; “metabolically unhealthy”) seem to have a higher risk of cardiovascular disease than their non-hyperinsulinemic (“metabolically healthy”) overweight counterparts. If certain combinations of metabolic health status and body size (“metabolically defined body size phenotypes”) are also associated with colorectal cancer, measurement of insulin levels in conjunction with body fat (adiposity) measurements such as body mass index (BMI; an indicator of body fat calculated by dividing a person’s weight in kilograms by their height in meters squared) might improve colorectal cancer risk assessment. In this nested case–control study, the researchers assess the associations between metabolically defined body size phenotypes and colorectal cancer risk. A nested case–control study identifies everyone in a group (here, participants in the European Prospective Investigation into Cancer and Nutrition [EPIC] study) who has a specific condition, identifies matched individuals in the same group without the condition, and asks whether these controls and the cases differ in terms of a specific characteristic or outcome.

**What Did the Researchers Do and Find?:**

The researchers matched 737 participants in the EPIC study who developed colorectal cancer after study enrollment with 737 controls and used serum concentrations of C-peptide, a marker of insulin secretion, and BMI measurements to classify each individual as metabolically healthy/normal weight, metabolically healthy/overweight, metabolically unhealthy/normal weight, or metabolically unhealthy/overweight. Specifically, the researchers categorized people as metabolically unhealthy if they had a C-peptide level above an arbitrarily chosen cut-off value based on the distribution of C-peptide levels in the control participants and as overweight if they had a BMI of ≥25 kg/m^2^ (the standard definition of overweight). Compared to metabolically healthy normal weight individuals, metabolically unhealthy normal weight and overweight individuals had an increased colorectal cancer risk; metabolically healthy overweight individuals had a similar colorectal cancer risk to metabolically healthy normal weight individuals. Among overweight individuals, metabolically healthy individuals had a lower colorectal cancer risk than metabolically unhealthy individuals. Finally, similar associations were seen when the researchers used waist circumference instead of BMI as the measure of adiposity.

**What Do These Findings Mean?:**

These findings suggest that normal weight individuals with hyperinsulinemia (the metabolically unhealthy normal weight phenotype) have a higher risk of colorectal cancer than normal weight individuals without hyperinsulinemia. They also suggest that metabolically unhealthy overweight individuals have a higher risk of colorectal cancer than metabolically healthy overweight individuals. The accuracy of these findings may be limited by the method the researchers used to classify individuals as hyperinsulinemic—there is no universally accepted clinical definition for using C-peptide level to diagnose hyperinsulinemia. Nevertheless, these findings suggest that the assessment of insulin levels in conjunction with adiposity measures might be a better way to assess an individual’s colorectal cancer risk than simply measuring adiposity, and might help to identify those individuals at high risk of colorectal cancer who are most likely to benefit from targeted interventions designed to prevent the onset of clinical disease.

**Additional Information:**

This list of resources contains links that can be accessed when viewing the PDF on a device or via the online version of the article at http://dx.doi.org/10.1371/journal.pmed.1001988.

- The US National Cancer Institute provides information for patients about all aspects of colorectal cancer; it also provides more detailed information colorectal cancer for health professionals and information on cancer risk and obesity
- The UK National Health Service Choices website has information and personal stories about colorectal cancer and information on obesity
- The not-for-profit organization Cancer Research UK provides information about colorectal cancer and about the association between cancer and obesity
- MedlinePlus provides links to further resources about colorectal cancer and about obesity
- Wikipedia has a page on hyperinsulinemia (note that Wikipedia is a free online encyclopedia that anyone can edit; available in several languages)
- More information about the EPIC study is available

## Introduction

Obesity has been consistently associated with increased risks of certain chronic diseases, such as cardiovascular disease (CVD), type 2 diabetes (T2D), and cancer [1–4]. High body mass index (BMI) and several other measures of adiposity have been consistently and strongly associated with colorectal cancer. In the European Prospective Investigation into Cancer and Nutrition (EPIC), men and women in the highest quintile of waist circumference had a 40% and 50% higher risk, respectively, of developing colon cancer compared to those in the lowest quintile [5]. A meta-analysis of 30 cohort studies reported elevated risks (relative risks) for those categorised as overweight (25–29.9 kg/m^2^) and obese (≥30 kg/m^2^) of 1.13 (95% CI 1.06–1.19) and 1.31 (95% CI 1.19–1.45), respectively [2].

Hyperinsulinaemia and insulin resistance are commonly present in obese individuals and have been hypothesised to play a role in the aetiology of colorectal cancer [6]. For instance, higher circulating insulin levels have been previously associated with greater colorectal cancer risk [7,8]. Other studies have assessed insulin resistance by measuring the homeostatic model assessment index of insulin resistance (HOMA_IR_) or levels of C-peptide, which has a longer half-life than insulin and is considered a valid biomarker of pancreatic insulin secretion [9]. C-peptide levels have also generally been positively associated with colorectal cancer risk [10–12].

However, for CVD, T2D, and breast cancer, accumulating evidence has identified a sub-group of metabolically healthy overweight/obese individuals without hyperinsulinaemia who are seemingly at lower risk than their hyperinsulinaemic, metabolically unhealthy/overweight counterparts [13–15]. Similarly, normal-weight individuals have been subdivided into an “at risk” phenotype based on the prevalence of hyperinsulinaemia; individuals with this phenotype have been shown to exhibit elevated CVD, T2D, and breast cancer risks compared to their “low risk” normal-weight equivalents without hyperinsulinaemia [13–16].

To our knowledge, no prospective studies have investigated the association of metabolically defined body size phenotypes with colorectal cancer risk. The identification of sub-types of body size that are associated with colorectal cancer may be useful for risk stratification and further understanding of the pathophysiological mechanisms underlying the obesity–colorectal cancer relationship. Therefore, in this nested case–control analysis within the EPIC prospective cohort, we classified individuals into metabolically defined body size phenotype groups based on the presence or absence of hyperinsulinaemia (based on C-peptide level) combined with anthropometric measurements. The associations of these metabolically defined body size phenotypes with incident colorectal cancer were then assessed.

## Methods

### EPIC Study Population and Collection of Blood Samples

All study participants provided written informed consent. Ethical approval for the EPIC study was obtained from the review boards of the International Agency for Research on Cancer and local participating centres: National Committee on Health Research Ethics (Denmark); Comité de Protection des Personnes (France); Ethics Committee of the Heidelberg University Medical School (Germany); Ethikkommission der Landesärztekammer Brandenburg Cottbus (Germany); University of Athens Medical School (Greece) Comitato Etico Indipendente, Fondazione IRCCS Istituto Nazionale dei Tumori (Italy); Human Genetics Foundation Torino Ethics Committee (Italy); Medical Ethical Committee (METC) of the University Medical Center Utrecht (the Netherlands); Regional Ethical Committee for Northern Norway and the Norwegian Data Inspectorate (Norway); Comité de Ética de Investigación Clínica (Spain); Ethics Committee of Lund University (Sweden); Umea Regional Ethical Review Board (Sweden); Norwich District Ethics Committee (UK); Scotland A Research Ethics Committee (UK); and the Imperial College Research Ethics Committee (UK). EPIC is an ongoing multicentre prospective cohort study designed to investigate the associations between diet, lifestyle, and genetic and environmental factors and various types of cancer. A detailed description of the methods of the EPIC study has previously been published [17,18]. In summary, 521,448 participants (~70% women) mostly aged 35 y or above were recruited between 1992 and 2000. Participants were recruited from 23 study centres in ten European countries. The present study includes participants from Denmark, France, Germany, Greece, Italy, the Netherlands, Spain, and the United Kingdom. Blood samples were collected at baseline according to standardised procedures [17,18] and stored at the International Agency for Research on Cancer (−196°C, liquid nitrogen) for all countries except Denmark (−150°C, nitrogen vapour).

### Follow-Up for Cancer Incidence and Vital Status

Incident cancer cases were identified using population cancer registries in Denmark, Italy, the Netherlands, Spain, and the United Kingdom. In France, Germany, and Greece, cancer cases were identified during follow-up from a combination of sources including health insurance records, cancer and pathology registries, and active follow-up directly through study participants or their next of kin. The end of follow-up for the current study was defined as the latest date of complete follow-up (of whole cohort) for both cancer incidence and vital status; this ranged from December 1999 to June 2003 for centres using registry data and from June 2000 to December 2002 for centres that used active follow-up procedures. Colorectal cancer cases were defined using the tenth revision of the International Classification of Diseases (ICD-10) and the second revision of the International Classification of Diseases for Oncology (ICDO-2). Cancer of the colon included cancers within the caecum, appendix, ascending colon, hepatic flexure, transverse colon, splenic flexure, descending colon, sigmoid colon, overlapping sites of colon, and unspecified sites within the colon (C18.0–18.9). Cancer of the rectum included cancer occurring at the rectosigmoid junction (C19) and rectum (C20).

### Selection of Case and Control Participants

The current analysis uses data from a nested case–control study design in which serum C-peptide level was measured in 1,078 incident colorectal cancer cases and 1,078 matched controls from eight EPIC countries (excluding Norway and Sweden) [11]. Participants from Norway were not selected because the time period between blood collection and the laboratory analyses was too short for a sufficient number of colorectal cancer cases to accrue. Cases from Sweden were not included because an independent study of insulin and colorectal cancer risk within that population was ongoing when the laboratory analyses were undertaken. Controls were selected from the full cohort of individuals who were alive and free of cancer (except non-melanoma skin cancer) at the time of diagnosis of the cases, using incidence density sampling and with controls matched to cases by age (±6 mo at recruitment), sex, study centre, follow-up time since blood collection, time of day at blood collection (±4 h), fasting status, menopausal status, and phase of menstrual cycle at blood collection. Exclusion criteria for the current analysis included the following: individuals with diabetes (self-reported at baseline) or those with unknown diabetic status, individuals without information on fasting status when blood was collected, and women who reported using menopausal hormone therapy or oral contraceptives at the time of blood collection, due to the effect of exogenous hormone use on C-peptide levels, which may render the observed associations in hormone users uninterpretable [7]. After these exclusions, a total of 737 incident colorectal cancer cases and 737 matched controls with available baseline information were included in the analysis.

### Assessment of Anthropometric, Lifestyle, and Dietary Exposures

With participants not wearing shoes, weight was measured to the nearest 0.1 kg and height was measured—dependent on the study centre—to the nearest 0.1, 0.5, or 1.0 cm. BMI was calculated as weight in kilograms divided by height in metres squared (kg/m^2^). Waist circumference was measured either at the narrowest torso circumference or at the midpoint between the lower ribs and iliac crest. Lifestyle questionnaires were used to obtain information on education, smoking status, alcohol consumption, and physical activity level. Dietary information (dietary intake of total energy, red and processed meats, and fibre, calcium, and fish) was collected at baseline using validated country/centre-specific dietary questionnaires [17,18].

### Laboratory Measurements

C-peptide was assayed in serum samples of all participants (radioimmunoassay; Diagnostic System Laboratories) as previously described [11]. The mean intra-batch and inter-batch coefficients of variation were 4.6% and 7.5%, respectively, for C-peptide (at a concentration of 5 ng/ml) [11]. Levels of previously measured glycated haemoglobin (HbA1c) were also available for the majority of participants [19].

### Metabolically Defined Body Size Phenotype Definitions

Participants were divided into tertiles based on the distribution of C-peptide concentration amongst the control population (tertile cut-points: 2.96 ng/ml and 4.74 ng/ml), and were classified as metabolically healthy if below the first tertile of C-peptide and metabolically unhealthy if above the first tertile. These metabolic health definitions were then combined with BMI or waist circumference measurements to create four metabolic health/body size phenotype categories: (1) metabolically healthy/normal weight (BMI < 25 kg/m^2^ or waist circumference < 80 cm for women and < 94 cm for men), (2) metabolically healthy/overweight (BMI ≥ 25 kg/m^2^ or waist circumference ≥ 80 cm for women and ≥ 94 cm for men), (3) metabolically unhealthy/normal weight (BMI < 25 kg/m^2^ or waist circumference < 80 cm for women and < 94 cm for men), and (4) metabolically unhealthy/overweight (BMI ≥ 25 kg/m^2^ or waist circumference ≥ 80 cm for women and ≥ 94 cm for men). The International Diabetes Federation (IDF) waist circumference cut-points were used [20]; these are ethnic-specific cut-points for European populations.

### Statistical Analysis

Differences between cases and controls were assessed using the Wilcoxon two-sample test or two-sample *t*-test for continuous variables and the χ^2^ test for categorical variables. Conditional logistic regression, stratified by case–control set, was used to compute odds ratios (ORs) and 95% confidence intervals for the associations between metabolic-health-defined body size phenotypes and colorectal cancer, colon cancer, and rectal cancer. The basic model (model 1) was conditioned on including the matching criteria only, while the multivariable models (models 2 and 3) included the matching criteria plus additional adjustment for a set of a priori defined colorectal cancer risk factors that included smoking status, physical activity, education level, alcohol consumption, height, and dietary intakes of total energy, red and processed meats, and fibre. Further adjustment for dietary intakes of calcium and fish resulted in virtually unchanged risk estimates, so these two variables were excluded from the multivariable models. Models were additionally stratified by sex and formally tested for heterogeneity using χ^2^ tests. Heterogeneity between colon and rectal cancer was tested using χ^2^ tests. To assess whether preclinical disease may have influenced the results, cases diagnosed within the first 2 y of follow-up were excluded and all analyses were redone. In sensitivity analyses, all models were rerun (1) with a BMI cut-point of 30 kg/m^2^ (rather than 25 kg/m^2^) for metabolic health/body size phenotype definitions and (2) with participants who had HbA1c measurements > 6.5% (the recommended cut-point for diagnosing diabetes) excluded. Tests of interaction (multiplicative) between the dichotomous body size (BMI or waist circumference) and C-peptide variables used to define the metabolic health/body size phenotypes were assessed in separate models. The statistical significance of these interaction terms was assessed by conducting likelihood ratio tests on models with and without these cross-product terms. Statistical tests used in the analysis were all two-sided, and a *p*-value of <0.05 was considered statistically significant. Analyses were conducted using Stata v11.0.

## Results

Colorectal cancer cases had greater waist circumference measurements than controls (Table 1). A higher proportion of the controls were never smokers and physically active compared to the case participants. Control participants reported lower consumption of red and processed meats and had lower levels of serum C-peptide than cases. The median follow-up time was shorter for colon cancer cases (3.7 y) than for rectal cancer cases (3.9 y). Compared to the metabolically healthy/normal weight group, a greater proportion of metabolically unhealthy/normal weight participants were physically inactive and a lower proportion never smoked (Table 2). Compared to the metabolically unhealthy/overweight group, individuals in the metabolically healthy/overweight group were less likely to be current smokers and to be physically inactive, and they consumed less red and processed meats.

**Table 1:** Baseline characteristics of cases and controls.

Baseline Characteristic	Cases	Controls	*p*-Value*
**Cancer type, *n* (percent)**			
Colorectal cancer	737 (100.0%)	737 (100.0%)	
Colon cancer	444 (60.2%)	444 (60.2%)	
Rectal cancer	293 (39.8%)	293 (39.8%)	
**Sex, *n* (percent)**			
Men	395 (53.6%)	395 (53.6%)	
Women	342 (46.4%)	342 (46.4%)	
**Age at blood collection (years)** ^§^	57.6 (6.4)	57.6 (6.4)	0.96
**Years of follow-up** ^§^	3.7 (2.1)	—	
**Anthropometrics** ^§^			
BMI (kg/m^2^)	26.9 (4.3)	26.6 (3.8)	0.16
Waist circumference (cm)	91.2 (12.8)	89.8 (12.4)	0.03
**Smoking status** ^†^			0.56
Never	279 (37.9%)	306 (41.4%)	
Former	246 (33.4%)	229 (30.9%)	
Current	209 (28.4%)	202 (27.3%)	
**Physical activity** ^†^			0.06
Inactive	148 (20.1%)	121 (16.3%)	
Moderately inactive	218 (29.6%)	195 (26.3%)	
Moderately active	296 (40.2%)	332 (44.8%)	
Active	72 (9.8%)	85 (11.5%)	
**Education level** ^†^			0.77
None/primary school completed	297 (40.3%)	315 (42.5%)	
Technical/professional school	177 (24.0%)	183 (24.7%)	
Secondary school	105 (14.3%)	91 (12.3%)	
Longer education (including university degree)	146 (19.8%)	139 (18.8%)	
**Fasting status at blood collection** ^†^			1.00
Not fasting	374 (50.8%)	374 (50.8%)	
In between	155 (21.0%)	155 (21.0%)	
Fasting	208 (28.2%)	208 (28.2%)	
**Dietary intakes**			
Alcohol consumption (g/d)^‡^	11.8 (2.3–29.7)	12.0 (2.7–26.4)	0.27
Red and processed meats (g/d)^‡^	88.3 (58.2–122.7)	83.9 (52.2–121.8)	0.10
Fibre (g/d)^§^	23.3 (7.8)	23.9 (8.2)	0.13
Total energy (kcal/d)^‡^	2,150 (1,761–2,559)	2,131 (1,734–2,563)	0.44
**C-peptide (ng/ml)** ^‡^	3.9 (2.8–5.9)	3.7 (2.6–5.4)	0.01
**Metabolic health/BMI definition** ^a^			0.01
Metabolically healthy/normal weight	101 (13.7%)	131 (17.8%)	
Metabolically healthy/overweight	93 (12.6%)	121 (16.4%)	
Metabolically unhealthy/normal weight	158 (21.4%)	133 (18.0%)	
Metabolically unhealthy/overweight	385 (52.2%)	352 (47.8%)	
**Metabolic health/waist circumference definition** ^b^			0.004
Metabolically healthy/normal weight	113 (15.5%)	153 (20.9%)	
Metabolically healthy/overweight	80 (10.9%)	97 (13.3%)	
Metabolically unhealthy/normal weight	160 (21.9%)	168 (23.0%)	
Metabolically unhealthy/overweight	378 (51.7%)	313 (42.8%)	

For the metabolic health/BMI models, the category definitions are as follows: metabolically healthy/normal weight is individuals with normal BMI (<25 kg/m^2^) and below tertile 1 of C-peptide; metabolically healthy/overweight is individuals with overweight/obese BMI (≥25 kg/m^2^) plus below tertile 1 of C-peptide; metabolically unhealthy/normal weight is individuals with normal BMI (<25 kg/m^2^) plus above tertile 1 of C-peptide; metabolically unhealthy/overweight is individuals with overweight/obese BMI (≥25 kg/m^2^) plus above tertile 1 of C-peptide. The C-peptide tertile cut-points were 2.96 ng/ml and 4.74 ng/ml. For the metabolic health/IDF waist circumference models, the category definitions are as follows: metabolically healthy/normal weight is individuals with waist circumference below IDF cut-point (<80 cm in women; <94 cm in men) plus below tertile 1 of C-peptide; metabolically healthy/overweight is individuals with waist circumference above IDF cut-point (≥80 cm in women; ≥94 cm in men) plus below tertile 1 of C-peptide; metabolically unhealthy/normal weight is individuals with waist circumference below IDF cut-point (<80 cm in women; <94 cm in men) plus above tertile 1 of C-peptide; metabolically unhealthy/overweight is individuals with waist circumference above IDF cut-point (≥80 cm in women; ≥94 cm in men) plus above tertile 1 of C-peptide.

*Calculated using Wilcoxon two-sample test or two-sample *t*-test for continuous variables and χ2 test for categorical variables.

^§^Values are mean (standard deviation).

^†^Values are *n* (percent) with participants with any missing/unknown values for baseline characteristics excluded.

^‡^Values are median (interquartile range).

^a^Values are *n* (percent) based on 737 cases and 737 control participants.

^b^Values are *n* (percent) based on 731 cases and 731 control participants.

**Table 2:** Baseline characteristics of control group participants by metabolic health (hyperinsulinaemia)–defined body size phenotypes using body mass index or the International Diabetes Federation waist circumference cut-points.

Baseline Characteristic	Metabolic-Health-Defined Body Size Phenotype									
	Metabolic Health/BMI Definition					Metabolic Health/ IDF Waist Circumference Definition				
	Metabolically Healthy/Normal Weight (*n* = 131)^a^	Metabolically Healthy/Overweight (*n* = 121)^a^	Metabolically Unhealthy/Normal Weight (*n* = 133)^a^	Metabolically Unhealthy/Overweight (*n* = 352)^a^	*p*-Value	Metabolically Healthy/Normal Weight (*n* = 153)^a^	Metabolically Healthy/Overweight (*n* = 97)^a^	Metabolically Unhealthy/Normal Weight (*n* = 168)^a^	Metabolically Unhealthy/Overweight (*n* = 313)^a^	*p*-Value
**Age at blood collection (years)** ^§^	56.7 (6.4)	57.1 (6.5)	57.7 (6.7)	58.1 (6.3)	0.03	56.4 (6.6)	57.7 (6.1)	56.7 (6.8)	58.7 (6.0)	<0.001
**Anthropometrics** ^§^										
BMI (kg/m^2^)	22.7 (1.7)	27.8 (2.5)	23.0 (1.5)	29.0 (3.2)		23.5 (2.4)	27.6 (3.0)	24.1 (2.2)	29.1 (3.4)	
Waist circumference (cm)	77.4 (8.6)	91.3 (8.7)	81.5 (8.4)	97.0 (10.5)		78.1 (8.5)	93.5 (7.6)	82.0 (8.1)	93.4 (9.9)	
**Smoking status** ^†^					0.23					0.50
Never	59 (45.0%)	57 (47.1%)	44 (33.1%)	146 (41.5%)		73 (47.7%)	42 (43.3%)	64 (38.1%)	126 (38.8%)	
Former	32 (24.4%)	40 (33.1%)	45 (33.8%)	111 (31.5%)		38 (24.8%)	33 (34.0%)	51 (30.4%)	104 (32.8%)	
Current	39 (29.8%)	24 (19.8%)	44 (33.1%)	95 (27.0%)		41 (26.8%)	22 (22.7%)	53 (31.6%)	85 (26.8%)	
**Physical activity** ^†^					0.67					0.06
Inactive	17 (13.0%)	17 (14.1%)	25 (18.8%)	62 (17.7%)		21 (13.7%)	13 (13.4%)	36 (21.4%)	51 (16.3%)	
Moderately inactive	29 (22.1%)	31 (25.6%)	38 (28.6%)	97 (27.7%)		36 (23.5%)	23 (23.7%)	46 (27.4%)	87 (27.8%)	
Moderately active	65 (50.0%)	61 (50.4%)	52 (39.1%)	154 (44.0%)		73 (47.7%)	52 (53.6%)	56 (33.3%)	148 (47.3%)	
Active	18 (13.7%)	12 (9.9%)	18 (13.5%)	37 (10.6%)		21 (13.7%)	9 (9.3%)	27 (16.1%)	27 (8.6%)	
**Education level** ^†^					0.001					<0.001
None/primary school completed	35 (26.7%)	62 (51.2%)	46 (35.1%)	172 (48.6%)		42 (27.5%)	54 (55.7%)	59 (35.1%)	158 (50.5%)	
Technical/professional school	39 (30.0%)	21 (17.4%)	35 (26.7%)	88 (24.9%)		45 (29.4%)	14 (14.4%)	50 (30.0%)	72 (23.0%)	
Secondary school	24 (18.3%)	13 (10.7%)	16 (12.2%)	38 (10.7%)		26 (17.0%)	11 (11.3%)	20 (11.9%)	33 (10.5%)	
Longer education (including university degree)	31 (23.7%)	22 (18.2%)	34 (26.0%)	52 (14.7%)		37 (24.2%)	16 (16.5%)	37 (22.0%)	48 (15.3%)	
**Dietary intakes**										
Alcohol consumption (g/d)^‡^	12.2 (2.7–27.3)	12.0 (1.8–26.6)	11.5 (3.2–25.1)	12.0 (2.5–26.6)	0.43	12.2 (1.7–26.4)	12.0 (2.6–29.7)	10.2 (2.8–24.6)	12.3 (3.3–26.6)	0.32
Red and processed meats (g/d)^‡^	68.2 (44.9–102.6)	74.8 (52.0–107.5)	84.0 (52.0–122.1)	92.9 (56.8–130.8)	<0.001	76.1 (45.6–103.1)	70.0 (50.2–106.4)	84.9 (53.8–122.0)	92.9 (56.8–131.8)	<0.001
Fibre (g/d)^§^	23.1 (7.3)	23.6 (7.5)	25.0 (9.1)	24.0 (8.3)	0.31	23.4 (7.3)	23.5 (7.7)	24.9 (8.8)	24.0 (8.4)	0.37
Total energy (kcal/day)	2,084 (1,621–2,488)	2,054 (1,754–2,495)	2,121 (1,766–2,620)	2,176 (1,737–2,619)	0.09	2,142 (1,801–2,488)	1,907 (1,602–2,495)	2,157 (1,719–2,622)	2,171 (1,767–2,606)	0.16
**C-peptide (ng/ml)** ^‡^	2.1 (1.7–2.6)	2.3 (2.0–2.6)	4.6 (3.4–6.3)	4.8 (3.8–6.7)		2.2 (1.8–2.6)	2.3 (2.0–2.7)	4.6 (3.6–6.2)	4.8 (3.8–6.8)	

For the metabolic health/BMI models, the category definitions are as follows: metabolically healthy/normal weight is individuals with normal BMI (<25 kg/m^2^) plus below tertile 1 of C-peptide; metabolically healthy/overweight is individuals with overweight/obese BMI (≥25 kg/m^2^) plus below tertile 1 of C-peptide; metabolically unhealthy/normal weight is individuals with normal BMI (<25 kg/m^2^) plus above tertile 1 of C-peptide; metabolically unhealthy/overweight is individuals with overweight/obese BMI (≥25 kg/m^2^) plus above tertile 1 of C-peptide. The C-peptide tertile cut-points were 2.96 ng/ml and 4.74 ng/ml. For the metabolic health/IDF waist circumference models, the category definitions are as follows: metabolically healthy/normal weight is individuals with waist circumference below IDF cut-point (<80 cm in women; <94 cm in men) plus below tertile 1 of C-peptide; metabolically healthy/overweight is individuals with waist circumference above IDF cut-point (≥80 cm in women; ≥94 cm in men) plus below tertile 1 of C-peptide; metabolically unhealthy/normal weight is individuals with waist circumference below IDF cut-point (<80 cm in women; <94 cm in men) plus above tertile 1 of C-peptide; metabolically unhealthy/overweight is individuals with waist circumference above IDF cut-point (≥80 cm in women; ≥94 cm in men) plus above tertile 1 of C-peptide.

^a^Number of control participants in the colorectal cancer models.

^§^Values are mean (standard deviation).

^†^Values are *n* (percent) with participants with missing/unknown values for baseline characteristics excluded.

^‡^Values are median (interquartile range).

### Metabolically Healthy/Overweight

#### Categorisation based on body mass index

Individuals with the metabolically healthy/overweight phenotype were not at elevated risk of colorectal cancer compared to metabolically healthy/normal weight individuals (OR = 0.96, 95% CI 0.65–1.42) (Table 3). In a sensitivity analysis, a similar null colorectal cancer relationship was observed when the BMI cut-point of 30 kg/m^2^ was used (rather than 25 kg/m^2^) (OR = 1.09, 95% CI 0.51–2.35). Individuals classified as metabolically healthy/overweight were at lower colorectal cancer risk than their metabolically unhealthy/overweight counterparts (OR = 0.69, 95% CI 0.49–0.96) (Table 3). No statistically significant heterogeneity was observed when colon cancer and rectal cancer were compared (*p* for heterogeneity = 0.47), and when men and women were analysed separately (*p* for heterogeneity = 0.17).

**Table 3:** Risk of colorectal cancer incidence associated with metabolic health (hyperinsulinaemia)–defined body size phenotypes using body mass index or the International Diabetes Federation waist circumference cut-points.

Model	Metabolic-Health-Defined Body Size Phenotype							
	Metabolic Health/BMI Definition				Metabolic Health/IDF Waist Circumference Definition			
	Metabolically Healthy/Normal Weight	Metabolically Healthy/Overweight	Metabolically Unhealthy/Normal Weight	Metabolically Unhealthy/Overweight	Metabolically Healthy/Normal Weight	Metabolically Healthy/Overweight	Metabolically Unhealthy/Normal Weight	Metabolically Unhealthy/Overweight
**Colorectal cancer**								
*N* cases/controls	101/131	93/121	158/133	385/352	113/153	80/97	160/168	378/313
Model 1	1.00	0.95 (0.65–1.40)	1.58 (1.11–2.25)	1.47 (1.07–2.00)	1.00	1.11 (0.74–1.65)	1.31 (0.94–1.83)	1.74 (1.28–2.37)
Model 2	1.00	0.96 (0.65–1.42)	1.59 (1.10–2.28)	1.40 (1.01–1.94)	1.00	1.12 (0.74–1.69)	1.35 (0.95–1.91)	1.66 (1.20–2.28)
Model 3	—	0.69 (0.49–0.96)	—	1.00	—	0.67 (0.47–0.97)	—	1.00
**Colon cancer**								
*N* cases/controls	59/73	57/85	88/80	240/206	69/91	46/67	86/105	240/178
Model 1	1.00	0.83 (0.51–1.34)	1.48 (0.93–2.38)	1.68 (1.10–2.58)	1.00	0.88 (0.53–1.48)	1.11 (0.71–1.74)	2.15 (1.42–3.24)
Model 2	1.00	0.87 (0.52–1.45)	1.49 (0.92–2.43)	1.75 (1.11–2.77)	1.00	0.90 (0.53–1.53)	1.18 (0.74–1.88)	2.12 (1.38–3.27)
Model 3	—	0.50 (0.32–0.77)	—	1.00	—	0.42 (0.26–0.70)	—	1.00
**Rectal cancer**								
*N* cases/controls	42/58	36/36	70/53	145/146	44/62	34/30	74/63	138/135
Model 1	1.00	1.34 (0.70–2.55)	1.78 (1.04–3.06)	1.31 (0.82–2.09)	1.00	1.61 (0.83–3.13)	1.64 (0.98–2.77)	1.42 (0.88–2.30)
Model 2	1.00	1.37 (0.70–2.68)	1.82 (1.02–3.23)	1.24 (0.76–2.02)	1.00	1.71 (0.85–3.44)	1.76 (1.01–3.05)	1.36 (0.82–2.26)
Model 3	—	1.11 (0.63–1.95)	—	1.00	—	1.26 (0.70–2.27)	—	1.00

Values are OR (95% CI). Model 1 was conditioned on matching factors only. Model 2 was conditioned on matching factors, with additional adjustment for height, smoking status, physical activity, education level, alcohol consumption, and dietary intakes of total energy, red and processed meats, and fibre. Model 3 was conditioned on matching factors, with additional adjustment for height, smoking status, physical activity, education level, alcohol consumption, and dietary intakes of total energy, red and processed meats, and fibre, among overweight participants only—with the metabolically unhealthy/overweight group as the reference category. For the metabolic health/BMI models, the category definitions are as follows: metabolically healthy/normal weight is individuals with normal BMI (<25 kg/m^2^) plus below tertile 1 of C-peptide; metabolically healthy/overweight is individuals with overweight/obese BMI (≥25 kg/m^2^) plus below tertile 1 of C-peptide; metabolically unhealthy/normal weight is individuals with normal BMI (<25 kg/m^2^) plus above tertile 1 of C-peptide; metabolically unhealthy/overweight is individuals with overweight/obese BMI (≥25 kg/m^2^) plus above tertile 1 of C-peptide. The C-peptide tertile cut-points were 2.96 ng/ml and 4.74 ng/ml. For the metabolic health/IDF waist circumference models, the category definitions are as follows: metabolically healthy/normal weight is individuals with waist circumference below IDF cut-point (<80 cm in women; <94 cm in men) plus below tertile 1 of C-peptide; metabolically healthy/overweight is individuals with waist circumference above IDF cut-point (≥80 cm in women; ≥94 cm in men) plus below tertile 1 of C-peptide; metabolically unhealthy/normal weight is individuals with waist circumference below IDF cut-point (<80 cm in women; <94 cm in men) plus above tertile 1 of C-peptide; metabolically unhealthy/overweight is individuals with waist circumference above IDF cut-point (≥80 cm in women; ≥94 cm in men) plus above tertile 1 of C-peptide.

#### Categorisation based on waist circumference

When waist circumference cut-points were used to categorise participants, metabolically healthy/overweight participants were, once more, at lower risk of colorectal cancer risk (OR = 0.67, 95% CI 0.47–0.97) than the metabolically unhealthy/overweight group, and not at higher risk than metabolically healthy/normal weight individuals (Table 3). There was no statistically significant difference in the associations when colon cancer and rectal cancer were compared (*p* for heterogeneity = 0.25), and when men and women were analysed separately (*p* for heterogeneity = 0.19).

### Metabolically Unhealthy/Normal Weight

#### Categorisation based on body mass index

Higher colorectal cancer risk (OR = 1.59, 95% CI 1.10–2.28) was observed amongst metabolically unhealthy/normal weight participants than among their metabolically healthy/normal weight counterparts (Table 3). This positive association persisted following additional adjustment for waist circumference (OR = 1.52, 95% CI 1.05–2.20). There was no statistically significant difference in the associations for rectal cancer compared to colon cancer (*p* for heterogeneity = 0.50) or by sex (*p* for heterogeneity = 0.26).

#### Categorisation based on waist circumference

Non-significantly higher colorectal cancer risk was observed for metabolically unhealthy/normal weight participants compared to metabolically healthy/normal weight participants when IDF waist circumference cut-points (≥80 cm in women and ≥94 cm in men) were used as the marker of adiposity (Table 3). No statistically significant heterogeneity was observed when men and women were analysed separately (*p* for heterogeneity = 0.22). When compared versus the metabolically healthy/normal weight group, a statistically significant positive association was observed for metabolically unhealthy/normal weight participants for rectal cancer (OR = 1.76, 95% CI 1.01–3.05) but not for colon cancer (OR = 1.18, 95% CI 0.74–1.88), although this difference in association for rectal versus colon cancer was non-significant (*p* for heterogeneity = 0.33).

### Metabolically Unhealthy/Overweight

#### Categorisation based on body mass index

Among the metabolically unhealthy/overweight group, higher colorectal cancer risk was observed compared with the metabolically healthy/normal weight individuals (OR = 1.40, 95% CI 1.01–1.94) (Table 3). No statistically significant heterogeneity in the relationship was observed for colon cancer and rectal cancer (*p* for heterogeneity = 0.47) or when men and women were analysed separately (*p* for heterogeneity = 0.32). A greater increased colon cancer risk was observed amongst the metabolically unhealthy/overweight group (OR = 1.75, 95% CI 1.11–2.77) than for overweight per se (i.e., when BMI was entered into the model as a dichotomous variable without consideration of hyperinsulinaemia; BMI ≥ 25 versus < 25 kg/m^2^, OR = 1.14, 95% CI 0.82–1.59).

#### Categorisation based on waist circumference

Higher colorectal cancer risk was observed among metabolically unhealthy/overweight individuals than among their metabolically healthy/normal weight counterparts (OR = 1.66, 95% CI 1.20–2.28) (Table 3). This positive relationship was statistically significant for colon cancer (OR = 2.12, 95% CI 1.38–3.27) but not for rectal cancer (OR = 1.36, 95% CI 0.82–2.26), although this difference in association for rectal versus colon cancer was non-significant (*p* for heterogeneity = 0.21). The positive colon cancer association for the metabolically unhealthy/overweight group was stronger than when waist circumference was entered into the model as a dichotomous variable without consideration of C-peptide level (≥80 cm women and ≥94 cm men versus <80 cm women and <94 cm men, OR = 1.58, 95% CI 1.14–2.19).

### Sensitivity Analyses

Exclusion of participants with HbA1c values > 6.5% (indicative of possible sub-clinical diabetes) did not lead to any appreciable change in the study results for any group versus metabolically healthy/normal weight based on BMI (metabolically healthy/overweight, OR = 0.98, 95% CI 0.65–1.48; metabolically unhealthy/normal weight, OR = 1.68, 95% CI 1.14–2.47; metabolically unhealthy/overweight OR = 1.35, 95% CI: 0.95–1.92) or waist circumference (metabolically healthy/overweight, OR = 1.08, 95% CI 0.70–1.68; metabolically unhealthy/normal weight, OR = 1.32, 95% CI 0.91–1.91; metabolically unhealthy/overweight, OR = 1.64, 95% CI 1.16–2.32). A similar pattern of results was observed when the first quartile or median C-peptide value, rather than the first tertile, was used to define metabolic health (hyperinsulinaemia) for the body size phenotypes (S1 Table). A similar pattern of results were observed when cases diagnosed within the first 2 y of follow-up were excluded (S2 Table). The *p*-interaction values between the dichotomous BMI and C-peptide variables used to define the metabolic health/body size phenotypes were as follows: colorectal cancer, *p* = 0.72; colon cancer, *p* = 0.35; and rectal cancer, *p* = 0.09. The *p*-interaction values between the dichotomous waist circumference and C-peptide variables used to define the metabolic health/body size phenotypes were as follows: colorectal cancer, *p* = 0.69; colon cancer, *p* = 0.03; and rectal cancer, *p* = 0.05.

## Discussion

The results of this prospective investigation indicate that normal-weight individuals with hyperinsulinaemia (the metabolically unhealthy/normal weight phenotype) are at higher colorectal cancer risk than those of normal-weight without hyperinsulinaemia. Our results also support the notion that metabolically healthy/overweight individuals, with normal insulin levels, are at reduced risk of colorectal cancer compared to their hyperinsulinaemic counterparts.

To our knowledge, this is the first investigation of hyperinsulinaemia-defined body size phenotypes and colorectal cancer risk in a prospective cohort setting. A number of studies have previously investigated the relationships of body size phenotypes with CVD, T2D, and breast cancer risks, and have reported elevated risks among metabolically unhealthy/normal weight individuals compared to their metabolically healthy/normal weight counterparts [13–16]. We observed a similar positive association for the metabolically unhealthy/normal weight phenotype when BMI and waist circumference were used as the anthropometric measure, and a statistically significant relationship was present only for rectal cancer and not for colon cancer. This result suggests that hyperinsulinaemia, independent of body size, may be a more relevant aetiological factor than adiposity per se; this is consistent with mitogenic and anti-apoptotic effects of insulin on the colon mucosa. Hypothesised causes of hyperinsulinaemia in normal-weight individuals, beyond an accumulation of visceral fat, may include low physical activity levels [21], low fibre intake [22], and changes in the actions of pro-inflammatory and anti-inflammatory cytokines [22,23].

In the metabolically healthy/overweight group, we observed no increased risk for colorectal cancer. This result is inconsistent with a recent Korean cross-sectional analysis that reported a 59% greater (OR 1.59, 95% CI 1.04–2.43) prevalence of high-risk colorectal adenoma for metabolically healthy/overweight individuals than for metabolically healthy/normal weight individuals [24]. In this previous analysis, the definition of metabolically unhealthy incorporated insulin resistance and metabolic syndrome criteria, such as blood pressure and abnormal levels of blood glucose and blood lipids. Within EPIC, when metabolic syndrome components and cut-points were analysed within the same multivariable model, only abnormal levels of blood glucose (as assessed by HbA1c measurements) were associated with colon cancer and rectal cancer [25]. These findings may reflect other potential mechanisms related to high abdominal fat accumulation being important for colorectal cancer risk, independent of hyperinsulinaemia. For example, visceral adipose tissue generates hormones and cytokines with inflammatory, metabolic, and direct carcinogenic potential, which may directly or indirectly increase colorectal cancer risk [26]. Therefore, potential pathways to explain this association include chronic low-grade inflammation and alterations in adipokine concentrations [26]. Future studies may shed more light on underlying pathophysiological mechanisms.

The increased colorectal cancer risk observed among the metabolically unhealthy/overweight group was present when both BMI and waist circumference measurements were used as markers of adiposity. Previous studies have shown a strong association between waist circumference and colon cancer [1,2,5]. In our analysis, a 58% greater risk of colon cancer was observed among participants above the IDF waist circumference cut-point (80 cm in women and 94 cm in men) compared to those below the cut-point. Interestingly, when individuals were subdivided into hyperinsulinaemia/body size phenotype groups, a higher risk estimate for the metabolically unhealthy/overweight group was observed (a 112% higher colon cancer risk). Overall, our results suggest that simply identifying those at greater risk of developing colorectal cancer by high BMI or waist circumference measurement would exclude normal-weight individuals with hyperinsulinaemia and underestimate the risk amongst overweight individuals with hyperinsulinaemia. Earlier identification of such individuals could lead to appropriate targeted interventions being introduced, which could prevent the onset of clinical disease.

A strength of our study is its prospective design, i.e., that pre-diagnostic measurements of C-peptide were used. Although the follow-up period was relatively short, a similar pattern of results was observed when cases with less than 2 y of follow-up were excluded. Our use of C-peptide level as a marker of insulin resistance, rather than the HOMA_IR_ (using insulin and glucose measures), was justified as C-peptide is a validated marker of hyperinsulinaemia [9] and has been previously associated with colorectal cancer risk [11,12]. A limitation is that the classification of individuals as hyperinsulinaemic—based on their C-peptide level—was arbitrary. However, when we used the first quartile or median of C-peptide, instead of the first tertile, as the cut-point of hyperinsulinaemia, a similar pattern of associations was observed. A possible limitation was that our study lacked statistical power for some of the sub-group analyses, e.g., for the analysis of metabolically healthy/overweight participants compared with the metabolically healthy/normal weight group; however, we estimated that we had 70% power (α = 0.05, two-sided test) to observe a similar relationship to what was found for the metabolically unhealthy/overweight group (OR 2.1). An additional potential limitation was that data on use of aspirin and other non-steroidal anti-inflammatory drugs (NSAIDs) that have been linked with reduced risk of colorectal cancer were not available for the majority of study participants and could therefore not be considered as a possible covariate in our multivariable models. However, we feel it is unlikely that aspirin or NSAID use would significantly confound the observed associations between hyperinsulinaemia-defined body size phenotypes and colorectal cancer, since previously reported associations of adiposity, C-peptide, and other hyperinsulinaemia parameters with colorectal cancer risk were unaffected by adjustment for aspirin or NSAID use [7,12,27,28].

Our results indicate that sub-classifying populations by hyperinsulinaemia and adiposity measurements could identify differential colorectal cancer risk relationships for the defined metabolic health/body size phenotypes. Our results were supportive of individuals with the metabolically healthy/overweight phenotype being at lower colorectal cancer risk than those with hyperinsulinaemia and suggest that the assessment of insulin level in conjunction with adiposity measures may be of greater value in the assessment of colorectal cancer risk than adiposity per se.

## Supporting Information

S1 Plan Prospective analysis plan. (DOC)

S1 STROBE Checklist STROBE statement. (DOC)

S1 Table Risk of colon cancer and rectal cancer incidence associated with metabolic-health-defined body size phenotypes using body mass index or the International Diabetes Federation waist circumference cut-points. Values are OR (95% CI). Cut-point of hyperinsulinaemia: first quartile (A) or median (B) of C-peptide.
(DOCX)

S2 Table Risk of colorectal cancer incidence associated with metabolic health (hyperinsulinaemia)–defined body size phenotypes using body mass index or the International Diabetes Federation waist circumference cut-points, with colorectal cancer cases diagnosed during the first 2 y of follow-up excluded (*n* = 209). Values are OR (95% CI).
(DOCX)

## Abbreviations

BMI: body mass index
CVD: cardiovascular disease
EPIC: European Prospective Investigation into Cancer and Nutrition
HOMA_IR_: homeostatic model assessment index of insulin resistance
IDF: International Diabetes Federation
OR: odds ratio
T2D: type 2 diabetes
