# Supplementary material for: A Nested Case–Control Study of Metabolically Defined Body Size Phenotypes and Risk of Colorectal Cancer in the European Prospective Investigation into Cancer and Nutrition (EPIC)
Source: PLoS Med. 2016 Apr 5;13(4):e1001988. doi: 10.1371/journal.pmed.1001988 (PMC4821615; doi:10.1371/journal.pmed.1001988)
Supplement: S1 Plan — (DOC) [file pmed.1001988.s001.doc]

# Scientific Research Proposal Form

# European Investigation into Cancer and Nutrition and Chronic Disease (EPIC)

| May 2012 |
| --- |
| Colorectal Working Group |
|  |
| Gunter, Marc |
| m.gunter@imperial.ac.uk |
| Imperial College |
| Metabolically-defined Body Size Subtypes and Risk of Colorectal Cancer |

**Metabolically-defined Body Size Subtypes and Risk of Colorectal Cancer**

**Introduction**

Obesity is a major risk factor for colorectal cancer with obese individuals experiencing 1.5-fold greater risk of developing this malignancy compared to normal weight individuals (1). However, most obese individuals never develop colorectal cancer, indicating that the metabolic and physiologic changes that accompany obesity vary and may have different implications for disease risk. Indeed, accumulating evidence suggest that obesity can be sub-divided into distinct phenotypes, based on the prevalence of metabolic parameters such as hyperinsulinaemia and insulin resistance (evaluated using the HOMAIR index or circulating insulin or C-peptide levels), and that these phenotypes may be clinically relevant.

A subset of obese individuals, termed ‘metabolically benign/healthy obese’ (MBO), appear to have a low burden of adiposity-related cardiometabolic abnormalities despite excess weight and are hypothesized to be at lower risk of cardiovascular disease (CVD) than their “at-risk” obese counterparts (2). Consistent with this hypothesis, it has been reported that compared to their at-risk counterparts, metabolically benign obese individuals had a significantly lower incidence of CVD (RR = 0.71, 95% CI, 0.57-0.90) (2).

Insulin resistance has been hypothesized to underlie, in part, the association of obesity with various tumour types, including those of the colorectum. Insulin resistance and hyperinsulinemia are prevalent in obese patients and insulin, in addition to its metabolic effects, has promitotic and antiapoptotic activity that may be tumorigenic. In laboratory models, for example, high insulin levels have been shown to promote the development of aberrant crypt foci in the colon (which are posited to be colorectal cancer precursors), as well as the growth of colon cancer cells (3). Furthermore, overexpression of the insulin receptor (IR) can induce cell transformation *in vitro* (4), and human colorectal adenocarcinomas have been shown to express the IR at high levels, indicating that these cells may be sensitive to the growth effects of insulin (5).

Several epidemiologic studies have reported positive associations of insulin, or its metabolite, C-peptide, with development of colorectal cancer using prospectively collected serum specimens. For example, recent data from postmenopausal women enrolled in the Women’s Health Initiative demonstrate statistically significant positive relationships between fasting insulin levels and incidence of colorectal cancer (RR=1.73; 95% confidence interval (CI), 1.16–2.57; *P*trend = 0.005]) (6). In EPIC, similar relationships were observed following evaluation of the association of non-fasting C-peptide levels with risk of colorectal cancer (ORq5-q1=1.56, 95% CI, 1.16-2.09, Ptrend=<0.01) (7).

However, these prior studies were not sub-stratified to study the molecular risk factors for colorectal cancer among just the overweight/obese. Therefore, using existing data on serum C-peptide we propose to assess the association of metabolically-defined body size phenotypes with risk of incident colorectal cancer among EPIC participants.

**OBJECTIVES**

1. To investigate the association of incident colorectal cancer with metabolically-defined body size phenotypes we will create the following four exposure categories:
2. Normal weight individuals with normal C-peptide concentrations [metabolically healthy normal weight];
3. Overweight/obese individuals with normal C-peptide concentrations [metabolically healthy overweight/obese];
4. Normal weight individuals with high C-peptide concentrations [metabolically unhealthy normal weight];
5. Overweight/obese individuals with high C-peptide concentrations[metabolically unhealthy overweight/obese].­

**ANALYSIS PLAN**

*Study Population*

We propose to analyze baseline data from EPIC participants with C-peptide measurements that were measured as part of a previous C-peptide and colorectal cancer analysis (Jenab et al., 1,078 incident colorectal cancer cases, 1,078 matched controls)11;

*General Considerations*

In our analysis, the metabolically healthy normal weight phenotype (i) will be the reference group, against which the relative risk of incident colorectal cancer will be calculated for (ii) metabolically healthy overweight/obese; (iii) metabolically unhealthy normal weight; and (iv) metabolically unhealthy overweight/obese.

*Statistical Analysis*

Descriptive tables will summarize the characteristics of the non-case study subjects across metabolic health defined body size phenotypes, and will include demographic variables, risk factors for colorectal cancer, and median levels of C-peptide. The associations (odds ratio; OR; and 95% confidence interval) between the body size phenotypes and risk of colorectal cancer, colon cancer, and rectal cancer will be derived from conditional logistic regression modelling. The basic models will be conditioned on including the matching criteria only (age [±6 months at recruitment], sex, study centre, follow-up time since blood collection, time of day at blood collection [±4 hours], fasting status, menopausal status, and phase of menstrual cycle at blood collection). All multivariable models will include established colorectal cancer risk factors such as smoking status, physical activity, education level, alcohol consumption, height, and dietary intakes of total energy, red and processed meats, and fibre. Additional covariate adjustments for other colorectal cancer risk factors (such as intakes of folate, fish, and calcium) will be tested. These additional covariates will be included in the multivariable models if the risk estimates change appreciably (>10%) with their inclusion. Statistical tests used in the analysis will all be two-sided and a P-value of <0.05 will be considered statistically significant.

*Exposure*: Metabolically-defined body size phenotypes defined, using C-peptide concentrations.

*Outcome*: Incident colorectal cancer.

*Pertinent Variables:*

1. Diagnosis of colorectal cancer during follow-up
2. Age at enrollment
3. Education
4. Diabetes
5. HRT use
6. Oral contraceptive use
7. BMI (also weight)
8. Waist circumference
9. Height
10. Physical activity levels
11. Alcohol intake
12. Red meat intake
13. Processed meat intake
14. Fish intake
15. Fibre intake
16. Calcium intake
17. Folate intake
18. Total energy intake
19. Smoking status
20. C-peptide

Reference List

(1) Moghaddam AA, Woodward M, Huxley R. Obesity and Risk of Colorectal Cancer: A Meta-analysis of 31 Studies with 70,000 Events. Cancer Epidemiology Biomarkers & Prevention 2007;16(12):2533-47.

(2) Ogorodnikova AD, Kim M, McGinn AP, et al. Incident Cardiovascular Disease Events in Metabolically Benign Obese Individuals. Obesity 2012;20(3):651-9.

(3) Tran TT, Naigamwalla D, Oprescu AI, et al. Hyperinsulinemia, But Not Other Factors Associated with Insulin Resistance, Acutely Enhances Colorectal Epithelial Proliferation in Vivo. Endocrinology 2006;147(4):1830-7.

(4) Giorgino F, Belfiore A, Milazzo G, et al. Overexpression of Insulin Receptors in Fibroblast and Ovary Cells Induces a Ligand-Mediated Transformed Phenotype. Molecular Endocrinology 1991;5(3):452-9.

(5) Kiunga GA, Raju J, Sabljic N, et al. Elevated insulin receptor protein expression in experimentally induced colonic tumors. Cancer Letters 2004;211(2):145-53.

(6) Gunter MJ, Hoover DR, Yu H, et al. Insulin, Insulin-like Growth Factor-I, Endogenous Estradiol, and Risk of Colorectal Cancer in Postmenopausal Women. Cancer Research 2008;68(1):329-37.

(7) Jenab M, Riboli E, Cleveland RJ, et al. Serum C-peptide, IGFBP-1 and IGFBP-2 and risk of colon and rectal cancers in the European Prospective Investigation into Cancer and Nutrition. Int J Cancer 2007;121(2):368-76.
