# Supplementary material for: A Nested Case–Control Study of Metabolically Defined Body Size Phenotypes and Risk of Colorectal Cancer in the European Prospective Investigation into Cancer and Nutrition (EPIC)
Source: PLoS Med. 2016 Apr 5;13(4):e1001988. doi: 10.1371/journal.pmed.1001988 (PMC4821615; doi:10.1371/journal.pmed.1001988)
Supplement: S1 STROBE Checklist — (DOC) [file pmed.1001988.s002.doc]

**A Nested Case-Control Study of Metabolically Defined Body Size Phenotypes and Risk of Colorectal Cancer in the European Prospective Investigation into Cancer and Nutrition (EPIC)**

STROBE Statement—Checklist of items that should be included in reports of ***cohort studies***

|  | Item No | Recommendation |
| --- | --- | --- |
| **Title and abstract** | 1 | (*a*) Indicate the study’s design with a commonly used term in the title or the abstract *[paragraph 5]* |
|  |  | (*b*) Provide in the abstract an informative and balanced summary of what was done and what was found *[paragraphs 4-7]* |
| Introduction | | |
| Background/rationale | 2 | Explain the scientific background and rationale for the investigation being reported *[paragraphs 8-11]* |
| Objectives | 3 | State specific objectives, including any prespecified hypotheses *[paragraph 12]* |
| Methods | | |
| Study design | 4 | Present key elements of study design early in the paper  *[paragraphs 13-20]* |
| Setting | 5 | Describe the setting, locations, and relevant dates, including periods of recruitment, exposure, follow-up, and data collection  *[paragraphs 8-12]* |
| Participants | 6 | (*a*) Give the eligibility criteria, and the sources and methods of selection of participants. Describe methods of follow-up  *[paragraphs 13-16]* |
|  |  | (*b*) For matched studies, give matching criteria and number of exposed and unexposed *[paragraph 16]* |
| Variables | 7 | Clearly define all outcomes, exposures, predictors, potential confounders, and effect modifiers. Give diagnostic criteria, if applicable *[paragraphs 15, 17, 18, 19, 20]* |
| Data sources/ measurement | 8* | For each variable of interest, give sources of data and details of methods of assessment (measurement). Describe comparability of assessment methods if there is more than one group *[paragraphs 17, 18, 19]* |
| Bias | 9 | Describe any efforts to address potential sources of bias *[paragraph 20]* |
| Study size | 10 | Explain how the study size was arrived at *[paragraph 16]* |
| Quantitative variables | 11 | Explain how quantitative variables were handled in the analyses. If applicable, describe which groupings were chosen and why *[paragraphs 19, 20]* |
| Statistical methods | 12 | (*a*) Describe all statistical methods, including those used to control for confounding  *[paragraph 20]* |
|  |  | (*b*) Describe any methods used to examine subgroups and interactions *[paragraph 20]* |
|  |  | (*c*) Explain how missing data were addressed (N/A) |
|  |  | (*d*) If applicable, explain how loss to follow-up was addressed (N/A) |
|  |  | (*e*) Describe any sensitivity analyses *[paragraph 20]* |
| Results | | |
| Participants | 13* | (a) Report numbers of individuals at each stage of study—eg numbers potentially eligible, examined for eligibility, confirmed eligible, included in the study, completing follow-up, and analysed *[paragraph 16]* |
|  |  | (b) Give reasons for non-participation at each stage (N/A) |
|  |  | (c) Consider use of a flow diagram (N/A) |
| Descriptive data | 14* | (a) Give characteristics of study participants (eg demographic, clinical, social) and information on exposures and potential confounders *[paragraph 22]* |
|  |  | (b) Indicate number of participants with missing data for each variable of interest (N/A) |
|  |  | (c) Summarise follow-up time (eg, average and total amount) *[paragraphs 22]* |
| Outcome data | 15* | Report numbers of outcome events or summary measures over time *[paragraph 16]* |
| Main results | 16 | (*a*) Give unadjusted estimates and, if applicable, confounder-adjusted estimates and their precision (eg, 95% confidence interval). Make clear which confounders were adjusted for and why they were included *[paragraphs 23-32, Table 3]* |
|  |  | (*b*) Report category boundaries when continuous variables were categorized (N/A) |
|  |  | (*c*) If relevant, consider translating estimates of relative risk into absolute risk for a meaningful time period (N/A) |
| Other analyses | 17 | Report other analyses done—eg analyses of subgroups and interactions, and sensitivity analyses *[paragraph 32]* |
| Discussion | | |
| Key results | 18 | Summarise key results with reference to study objectives *[paragraphs 33-34]* |
| Limitations | 19 | Discuss limitations of the study, taking into account sources of potential bias or imprecision. Discuss both direction and magnitude of any potential bias *[paragraph 38]* |
| Interpretation | 20 | Give a cautious overall interpretation of results considering objectives, limitations, multiplicity of analyses, results from similar studies, and other relevant evidence *[paragraph 39]* |
| Generalisability | 21 | Discuss the generalisability (external validity) of the study results *[paragraphs 37, 39]* |
| Other information | | |
| Funding | 22 | Give the source of funding and the role of the funders for the present study and, if applicable, for the original study on which the present article is based *[via submission site]* |

*Give information separately for exposed and unexposed groups.

**Note:** An Explanation and Elaboration article discusses each checklist item and gives methodological background and published examples of transparent reporting. The STROBE checklist is best used in conjunction with this article (freely available on the Web sites of PLoS Medicine at http://www.plosmedicine.org/, Annals of Internal Medicine at http://www.annals.org/, and Epidemiology at http://www.epidem.com/). Information on the STROBE Initiative is available at http://www.strobe-statement.org.
