# Supplementary material for: A Nested Case–Control Study of Metabolically Defined Body Size Phenotypes and Risk of Colorectal Cancer in the European Prospective Investigation into Cancer and Nutrition (EPIC)
Source: PLoS Med. 2016 Apr 5;13(4):e1001988. doi: 10.1371/journal.pmed.1001988 (PMC4821615; doi:10.1371/journal.pmed.1001988)
Supplement: S1 Table — Values are OR (95% CI). Cut-point of hyperinsulinaemia: first quartile (A) or median (B) of C-peptide. (DOCX) [file pmed.1001988.s003.docx]

**S1 Table.** Risk of colon cancer and rectal cancer incidence associated with metabolic-health-defined body size phenotypes using body mass index or the International Diabetes Federation waist circumference cut-points. Values are OR (95% CI). Cut-point of hyperinsulinaemia: first quartile (A) or median (B) of C-peptide.

|  | **Metabolic-Health-Defined Body Size Phenotype** | | | | | | | | |
| --- | --- | --- | --- | --- | --- | --- | --- | --- | --- |
| **A** | **Metabolic Health/BMI Definition** | | | |  | **Metabolic Health/IDF Waist Circumference Definition** | | | |
| **C-peptide quartiles** | **Metabolically Healthy/Normal Weight** | **Metabolically Healthy/Overweight** | **Metabolically Unhealthy/Normal Weight** | **Metabolically Unhealthy/Overweight** |  | **Metabolically Healthy/Normal Weight** | **Metabolically Healthy/Overweight** | **Metabolically Unhealthy/Normal Weight** | **Metabolically Unhealthy/Overweight** |
| **Colon cancer** |  |  |  |  |  |  |  |  |  |
| Model 2 | 1.00 | 0.84 (0.46-1.51) | 1.25 (0.75-2.10) | 1.49 (0.91-2.44) |  | 1.00 | 0.81 (0.43-1.53) | 1.00 (0.61-1.63) | 1.82 (1.15-2.90) |
| Model 3^‡^ | - | 0.56 (0.34-0.92) | - | 1.00 |  | - | 0.44 (0.25-0.79) | - | 1.00 |
| **Rectal cancer** |  |  |  |  |  |  |  |  |  |
| Model 2 | 1.00 | 1.51 (0.72-3.17) | 1.80 (0.97-3.34) | 1.28 (0.75-2.18) |  | 1.00 | 1.82 (0.84-3.91) | 1.67 (0.94-2.98) | 1.39 (0.80-2.40) |
| Model 3^‡^ | - | 1.18 (0.64-2.15) | - | 1.00 |  | - | 1.31 (0.70-2.45) | - | 1.00 |
|  | **Metabolic-Health-Defined Body Size Phenotype** | | | | | | | | |
| **B** | **Metabolic Health/BMI Definition** | | | |  | **Metabolic Health/IDF Waist Circumference Definition** | | | |
| **C-peptide median** | **Metabolically Healthy/Normal Weight** | **Metabolically Healthy/Overweight** | **Metabolically Unhealthy/Normal Weight** | **Metabolically Unhealthy/Overweight** |  | **Metabolically Healthy/Normal Weight** | **Metabolically Healthy/Overweight** | **Metabolically Unhealthy/Normal Weight** | **Metabolically Unhealthy/Overweight** |
| **Colon cancer** |  |  |  |  |  |  |  |  |  |
| Model 2 | 1.00 | 1.08 (0.71-1.64) | 1.48 (0.87-2.50) | 1.59 (1.02-2.48) |  | 1.00 | 1.24 (0.80-1.91) | 1.07 (0.64-1.77) | 1.97 (1.29-3.01) |
| Model 3^‡^ | - | 0.68 (0.46-0.99) | - | 1.00 |  | - | 0.63 (0.41-0.96) | - | 1.00 |
| **Rectal cancer** |  |  |  |  |  |  |  |  |  |
| Model 2 | 1.00 | 1.28 (0.75-2.19) | 1.59 (0.86-2.95) | 1.03 (0.64-1.65) |  | 1.00 | 1.30 (0.73-2.30) | 1.26 (0.70-2.26) | 1.07 (0.64-1.78) |
| Model 3^‡^ | - | 1.24 (0.76-2.04) | - | 1.00 |  | - | 1.22 (0.73-2.02) | - | 1.00 |

Values are OR (95% CI). Model 2 was conditioned on matching factors, with additional adjustment for height, smoking status, physical activity, education level, alcohol consumption, and dietary intakes of total energy, red and processed meats, and fibre.
^‡^ Model 3 was conditioned on matching factors, with additional adjustment for height, smoking status, physical activity, education level, alcohol consumption, and dietary intakes of total energy, red and processed meats, and fibre, among overweight participants only - with metabolically unhealthy/overweight group as the reference category.

**A** For the metabolic health/BMI models, the category definitions are as follows: metabolically healthy/normal weight is individuals with normal BMI (<25 kg/m^2^) plus below quartile 1 of C-peptide; metabolically healthy/overweight is individuals with overweight/obese BMI (≥25 kg/m^2^) plus below quartile 1 of C-peptide; metabolically unhealthy/normal weight is individuals with normal BMI (<25 kg/m^2^) plus above quartile 1 of C-peptide; metabolically unhealthy/overweight is individuals with overweight/obese BMI (≥25 kg/m^2^) plus above quartile 1 of C-peptide. The C-peptide quartile cut-points are 2.62 ng/ml, 3.70 ng/ml, and 5.41 ng/ml.
For the metabolic health/IDF waist circumference models, the category definitions are as follows: metabolically healthy/normal weight is individuals with waist circumference below IDF cut-points (<80 cm in women; <94 cm in men) plus below quartile 1 of C-peptide; metabolically healthy/overweight is individuals with waist circumference above IDF cut-points (≥80 cm in women; ≥94 cm in men) plus below quartile 1 of C-peptide; metabolically unhealthy/normal weight is individuals with waist circumference below IDF cut-points (<80 cm in women; <94 cm in men) plus above quartile 1 of C-peptide; metabolically unhealthy/overweight is individuals with waist circumference above IDF cut-points (≥80 cm in women; ≥94 cm in men) plus above quartile 1 of C-peptide.

**B** For the metabolic health/BMI models, the category definitions are as follows: metabolically healthy/normal weight is individuals with normal BMI (<25 kg/m^2^) plus C-peptide below median; metabolically healthy/overweight is individuals with overweight/obese BMI (≥25 kg/m^2^) plus C-peptide below median; metabolically unhealthy/normal weight is individuals with normal BMI (<25 kg/m^2^) plus C-peptide above median; metabolically unhealthy/overweight is individuals with overweight/obese BMI (≥25 kg/m^2^) plus C-peptide above median. The C-peptide median cut-point was 3.70 ng/ml.
For the metabolic health/IDF waist circumference models, the category definitions are as follows: metabolically healthy/normal weight is individuals with waist circumference below IDF cut-points (<80 cm in women; <94 cm in men) plus C-peptide below median; metabolically healthy/overweight is individuals with waist circumference above IDF cut-points (≥80 cm in women; ≥94 cm in men) plus C-peptide below median; metabolically unhealthy/normal weight is individuals with waist circumference below IDF cut-points (<80 cm in women; <94 cm in men) plus C-peptide above median; metabolically unhealthy/overweight is individuals with waist circumference above IDF cut-points (≥80 cm in women; ≥94 cm in men) plus C-peptide above median.
