# Supplementary material for: A Nested Case–Control Study of Metabolically Defined Body Size Phenotypes and Risk of Colorectal Cancer in the European Prospective Investigation into Cancer and Nutrition (EPIC)
Source: PLoS Med. 2016 Apr 5;13(4):e1001988. doi: 10.1371/journal.pmed.1001988 (PMC4821615; doi:10.1371/journal.pmed.1001988)
Supplement: S2 Table — Values are OR (95% CI). (DOCX) [file pmed.1001988.s004.docx]

**S2 Table.** Risk of colorectal cancer incidence associated with metabolic health (hyperinsulinaemia)-defined body size phenotypes using body mass index or the International Diabetes Federation waist circumference cut-points, with colorectal cancer cases diagnosed during the first two years of follow-up excluded (*n* = 209). Values are OR (95% CI).

|  | **Metabolic-Health-Defined Body Size Phenotype** | | | | | | | | |
| --- | --- | --- | --- | --- | --- | --- | --- | --- | --- |
|  | **Metabolic Health/BMI Definition** | | | |  | **Metabolic Health/IDF Waist Circumference Definition** | | | |
|  | **Metabolically Healthy/Normal Weight** | **Metabolically Healthy/Overweight** | **Metabolically Unhealthy/Normal Weight** | **Metabolically Unhealthy/Overweight** |  | **Metabolically Healthy/Normal Weight** | **Metabolically Healthy/Overweight** | **Metabolically Unhealthy/Normal Weight** | **Metabolically Unhealthy/Overweight** |
| **Colorectal cancer** | |  |  |  |  |  |  |  |  |
| Model 2 | 1.00 | 0.93 (0.59-1.47) | 1.36 (0.88-2.10) | 1.13 (0.78-1.65) |  | 1.00 | 1.17 (0.72-1.90) | 1.10 (0.72-1.66) | 1.38 (0.96-2.01) |
| Model 3^‡^ | - | 0.82 (0.56-1.21) | - | 1.00 |  | - | 0.84 (0.55-1.28) | - | 1.00 |
| **Colon cancer** | |  |  |  |  |  |  |  |  |
| Model 2 | 1.00 | 0.90 (0.50-1.62) | 1.54 (0.85-2.80) | 1.61 (0.94-2.75) |  | 1.00 | 1.05 (0.56-1.99) | 1.18 (0.67-2.09) | 1.96 (1.18-3.25) |
| Model 3^‡^ | - | 0.56 (0.33-0.93) | - | 1.00 |  | - | 0.54 (0.30-0.97) | - | 1.00 |
| **Rectal cancer** | |  |  |  |  |  |  |  |  |
| Model 2 | 1.00 | 1.18 (0.53-2.63) | 1.23 (0.62-2.45) | 0.87 (0.49-1.55) |  | 1.00 | 1.25 (0.56-2.80) | 1.00 (0.51-1.93) | 0.96 (0.52-1.74) |
| Model 3^‡^ | - | 1.35 (0.70-2.59) | **-** | 1.00 |  | - | 1.31 (0.69-2.50) | **-** | 1.00 |

Values are OR (95% CI). Model 2 was conditioned on matching factors, with additional adjustment for height, smoking status, physical activity, education level, alcohol consumption, and dietary intakes of total energy, red and processed meats, and fibre.
^‡^ Model 3 was conditioned on matching factors, with additional adjustment for height, smoking status, physical activity, education level, alcohol consumption, and dietary intakes of total energy, red and processed meats, and fibre, among overweight participants only - with metabolically unhealthy/overweight group as the reference category.
For the metabolic health/BMI models, the category definitions are as follows: metabolically healthy/normal weight is individuals with normal BMI (<25 kg/m^2^) plus below tertile 1 of C-peptide; metabolically healthy/overweight is individuals with overweight/obese BMI (≥25 kg/m^2^) plus below tertile 1 of C-peptide; metabolically unhealthy/normal weight is individuals with normal BMI (<25 kg/m^2^) plus above tertile 1 of C-peptide; metabolically unhealthy/overweight is individuals with overweight/obese BMI (≥25 kg/m^2^) plus above tertile 1 of C-peptide. The C-peptide tertile cut-points were 2.96 ng/ml and 4.74 ng/ml. For the metabolic health/IDF waist circumference models, the category definitions are as follows: metabolically healthy/normal weight is individuals with waist circumference below IDF cut-points (<80 cm in women; <94 cm in men) plus below tertile 1 of C-peptide; metabolically healthy/overweight is individuals with waist circumference above IDF cut-points (≥80 cm in women; ≥94 cm in men) plus below tertile 1 of C-peptide; metabolically unhealthy/normal weight is individuals with waist circumference below IDF cut-points (<80 cm in women; <94 cm in men) plus above tertile 1 of C-peptide; metabolically unhealthy/overweight is individuals with waist circumference above IDF cut-points (≥80 cm in women; ≥94 cm in men) plus above tertile 1 of C-peptide.
